# Janus Kinase Signaling Pathway and Its Role in COVID-19 Inflammatory, Vascular, and Thrombotic Manifestations

**DOI:** 10.3390/cells11020306

**Published:** 2022-01-17

**Authors:** Jonathan D. Ravid, Orly Leiva, Vipul C. Chitalia

**Affiliations:** 1 Internal Medicine Residency Program, Cleveland Clinic, Cleveland, OH 44195, USA; jravid@gmail.com; 2 Division of Cardiology, Department of Medicine, New York University Langone Health, New York, NY 10016, USA; Orly.Leiva@nyulangone.org; 3 Renal Section, Department of Medicine, Boston University School of Medicine, Boston, MA 02139, USA; 4 Veterans Affairs Boston Healthcare System, Boston, MA 02118, USA; 5 Institute of Medical Engineering and Sciences, Massachusetts Institute of Technology, Cambridge, MA 02139, USA

**Keywords:** Janus kinase signaling, COVID-19, SARS-CoV-2, thrombosis, inflammation

## Abstract

Acute respiratory syndrome-coronavirus-2 (SARS-CoV-2) infection continues to be a worldwide public health crisis. Among the several severe manifestations of this disease, thrombotic processes drive the catastrophic organ failure and mortality in these patients. In addition to a well-established cytokine storm associated with the disease, perturbations in platelets, endothelial cells, and the coagulation system are key in triggering systemic coagulopathy, involving both the macro- and microvasculatures of different organs. Of the several mechanisms that might contribute to dysregulation of these cells following SARS-CoV-2 infection, the current review focuses on the role of activated Janus kinase (JAK) signaling in augmenting thrombotic processes and organ dysfunction. The review concludes with presenting the current understanding and emerging controversies concerning the potential therapeutic applications of JAK inhibitors for ameliorating the inflammation-thrombosis phenotype in COVID-19 patients.

## 1. Overview of Vascular, Thrombotic, and Inflammation Manifestations of Coronavirus Disease-19

Coronavirus Disease-19 (COVID-19) is a worldwide public health crisis caused by the severe acute respiratory syndrome coronavirus 2 (SARS-CoV-2). Beyond respiratory complications, such as acute respiratory distress syndrome (ARDS) cardiovascular, renal, and thrombotic complications contribute to catastrophic-end organ damage and multisystem organ failure driving the mortality associated with COVID-19 [1,2,3,4]. Macro- and microvascular complications have been observed in COVID-19 infection [1,4,5,6,7]. In the case of microvascular complications, direct infection of endothelial cells by SARS-CoV-2 and in-situ microvascular thrombosis were documented in several autopsy series [4,6,8]. Macrovascular complications, including venous thromboembolism (VTE) and arterial thrombosis have been described in several cohort and registry studies. In the CORONA-19-VTE registry, 30-day rates of major arterial or venous thromboembolic events were monitored in 1114 patients with COVID-19 in outpatient, non-intensive care unit (ICU) settings vs. in the ICU. These complications were found to be 17-fold higher in patients admitted to the ICU (35.3%) than in non-ICU (2.6%) or outpatient settings (0%). The CORONA-VTE registry also showed that myocardial infarction (MI) occurred in 7.7% of patients in the ICU setting and 0.5% of patients in the non-ICU setting [1]. Other studies have similarly found high rates of VTE among patients in the ICU [9,10,11]. Additionally, cerebrovascular events (CVA), including ischemic strokes, have been described as potential vascular complications in COVID-19 infection [12,13]. In a study of patients admitted for COVID-19 in Milan, Italy, stroke occurred in 2.5% of patients [14]. Importantly, thrombotic events in COVID-19 and SARS-CoV-1 involving large artery strokes occurred despite therapeutic anticoagulation and with relatively fewer vascular risk factors [15]. Additionally, acute lower limb ischemia and thrombosis in patients with COVID-19 infection have also been reported [16]. Further, patients with cardiovascular risk factors, such as hypertension, obesity, and diabetes, have higher risk of developing thrombotic complications [17,18]. 

A hyperinflammatory state is a hallmark of COVID-19. Similar to other epidemic coronaviruses (Middle East respiratory virus and SARS-CoV-1), COVID-19 begins with an initial phase of viral replication followed by an ensemble, inflammatory-driven second phase [19]. Indeed, serum levels of inflammatory mediators and markers, including interleukin (IL)-6, C-reactive protein (CRP), IL-1β, IL-10, CXCL9, CXCL10, ferritin, and tumor necrosis factor-α (TNF-α) are elevated in cases of COVID-19 [2,20,21]. Alveolar pneumocytes, epithelial cells and/or macrophages infected with SARS-CoV-2 release inflammatory cytokines such as IL-6 as well as chemokines, which further attract other circulating inflammatory cytokine-producing cells, such as neutrophils, natural killer cells, and T-lymphocytes [22]. All these events culminate in relentless feedback and feedforward loops exacerbating inflammation in patients with COVID-19. The degree of inflammation has prognostic significance in these patients, with levels of CRP and IL-6 being positively associated with increased risk of adverse events including mortality, thrombosis, and end organ injury [23,24]. The role of inflammation in driving COVID-19 morbidity and mortality has also been studied in clinical trials using anti-inflammatory therapies, including glucocorticoids and IL-6 inhibitors, and reporting their improving effects on mortality and organ complications in severe COVID-19 patients [25,26,27,28].

## 2. SARS-CoV-2 Entry into Target Cells

Among several potential surface receptors for entry of SARS-CoV-2 in target cells, such as CD209L/CD209 [29], the neuropilin receptor [30,31], and CD147/Basigin [32], the angiotensin-converting enzyme II (ACE2) is one well studied. ACE2 is responsible for the receptor-mediated endocytosis of SARS-CoV-2 into cells. A wide range of cells, including lung alveolar epithelial cells, renal epithelial cells, endothelial cells, cardiac myocytes, and immune cells, express ACE2 [33,34]. Macrophages and monocytes also express ACE2 receptors [35], as found in human spleen and lymph nodes of COVID-19 patients [36]. The cell surface abundance of the ACE2 receptor is controlled by clathrin-dependent endocytosis [34]. The viral spike protein S1 subunit facilitates binding to the ACE2 receptor, while the spike protein S2 subunit is cleaved by type II transmembrane serine protease (TMPRSS2), which allows viral entry into the cell. This event is followed by replication, cellular metabolic damage and release of inflammatory cytokines [37]. Interestingly, interferon (INF)-α2 was shown in different cell systems to upregulate the expression of ACE2 in a loop-back mechanism, leading to further augmentation in intracellular viral load [38]. Relevant to the current review, this effect is dependent on the Janus kinase (JAK) signaling cascade [39]. 

## 3. Janus Kinases Signaling towards Inflammation in COVID-19

**Figure 1:**
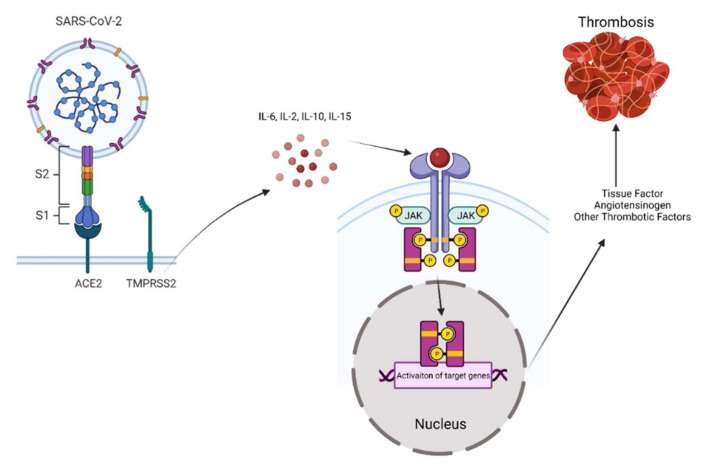
Schematic illustration of SARS-CoV-2-induced JAK–STAT activation and thrombosis.

Inflammation and cytokine storm contribute to a vasculo-thrombotic phenotype in COVID-19 patients [40]. Several of these cytokines signal through a receptor activation-induced JAK signaling cascade. There are four JAK members: JAK1, JAK2, JAK3 and TYK2, and seven signal transducer and activators of transcription (STAT) members: STAT1, STAT2, STAT3, STAT4, STAT5A, STAT5B, and STAT6 [41]. Activated JAKs tyrosine-phosphorylate the intracellular tails of the receptors, allowing the binding of members of the STAT family of transcription factors, with the latter being then phosphorylated by JAK, leading to disassociation of STAT from the receptor and translocation to the nucleus. Nuclear STAT controls the expression of genes encoding regulatory proteins involved in a variety of roles, such as cellular proliferation, differentiation, and immune response. Indeed, JAK–STAT signaling pathways have been shown to control several processes, such as aging, inflammation, and malignancy [42]. As reviewed in [43], an array of cytokines signal through the JAK–STAT pathway, including those elevated in COVID-19, such as IL-6, IL-2, IL-15, and IL-10. IL-6 binds to the soluble IL-6 receptor glycoprotein 130 to activate JAK–STAT signaling in various cells, which, in turn, releases chemokines and promotes monocyte and neutrophil recruitment [44]. IL-2 binding to natural killer (NK) cells activates JAK 1/3, leading to augmented NK cytotoxicity [45], while IL-15 activation of JAK1 in NK cells regulates their function. Further, the differentiation and lineage amplification of CD4+ and CD8+ T cells, which play a key role in eliminating SARS-CoV-2 from infected cells, is mediated by the JAK–STAT pathway [46]. Taken together, a wealth of literature implicates JAK–STAT in shaping an inflammatory milieu in COVID-19 patients. In view of the role of inflammatory cytokines in vasculothrombosis, it is reasonable to deduce that JAK–STAT signaling plays a role in the development of this aspect of COVID-19 pathology, as illustrated in Figure 1.

A cytokine storm associated with SARS-CoV-2 infection activates JAK–STAT signaling in target cells, including endothelial and inflammatory cells. In turn, activated JAK–STAT upregulates tissue factors and other thrombotic factors such as von Willebrand factor (vWF) to trigger an extrinsic coagulation cascade, contributing to wide spread macro and microvascular thrombosis.

## 4. The Role of JAK Signaling in Mediating the Effects of SARS-CoV-2 in Endothelial Cells, Coagulation, and Thrombosis

Thrombosis is a highly complex and dynamic process orchestrated by a myriad of cell types, including endothelial cells and platelets. Dysfunctional endothelial cells serve as a reactive vascular bed on which thrombosis is further propagated by platelets and other inflammatory cells. SARS-CoV-2 infection is considered a systemic endotheliopathy involving several vascular beds, considering its prominent vasculothrombotic manifestations [2,47,48]. The effects of SARS-CoV-2 on endothelial cells are mediated through receptor binding and/or through a SARS-CoV-2-induced inflammatory cytokine storm. Endothelial cells upon viral entry and replication eventually initiate a programmed cell death [49]. At the same time, pro-inflammatory cytokines produced by a host of cells, including bronchial epithelium and immune cells, activate JAK signaling in endothelial cells to further increase the production of inflammatory cytokines. This event in vascular endothelial cells also increases the production of von Willebrand factor (vWF) and angiopoietin-2, which further amplify the activation of vascular endothelial layers, as well as pulmonary endothelium [50]. Such activated endothelial cells release tissue factor (TF), which is the primary trigger of the extrinsic coagulation pathway, leading to the conversion of prothrombin to thrombin, and eventual clot formation. 

Additional JAK-regulated mechanisms could account for TF elevation and increased coagulopathy in COVID-19. Cells infected with coronaviruses eventually display suppressed levels of ACE-2 expression. This phenomenon increases the expression of angiotensin 2 (ANG-II) [51]. In turn, ANG-II is known to upregulate TF expression, and the propensity for microvascular thrombosis in COVID-19 [52]. Interestingly, past studies showed that the JAK–STAT pathway mediates the upregulation of ANG-II to control blood pressure [53]. Similarly, leukocytes activated through direct binding of SARS-CoV-2 or through the cytokine storm release TF into the blood and promote a procoagulant state. These results are consistent with the known role of JAK2–STAT3 signaling in regulating TF [54]. Additionally, SARS-CoV-2 can activate TF independent of JAK–STAT signaling. SARS-CoV-2-infected cells show increased surface TF activation through acid sphingomyelinase [55]. 

These mechanistic studies are in line with the changes in the extrinsic coagulation pathway observed in patients with COVID-19, such as modestly elevated prothrombin (PT), normal or suppressed aPTT, and elevated fibrinogen and D-dimer [56,57,58,59]. Collectively, the above studies support the notion that SARS-CoV-2-induced inflammatory cytokines activating various target cells, including endothelial cells, signal via the JAK–STAT pathway. Activated JAK–STAT, in turn, upregulates ANG-II, vWF, and TF, with consequent effects on the extrinsic coagulation cascade. Considering these observations, both TF [52] and the JAK–STAT pathway have been suggested as tantalizing therapeutic targets for thrombovascular complications in patients with COVID-19.

## 5. The Role of JAK Signaling in Mediating the Effects of SARS-CoV-2 in Platelets

The key roles platelets play in viral infection-mediated thrombosis has been previously described [60]. Similarly, the contribution of platelet activation to the pathophysiology of SARS-CoV-2 infection has been studied by several groups, as have the signaling mechanisms involved [29,61,62,63]. A prospective clinical study concluded that changes in platelet gene expression and activation properties contribute to severe thrombotic events in COVID-19 patients with severe clinical symptoms. Platelet profiling, such as with elevated surface P-selectin expression, platelet aggregation, adhesion, and spreading abilities, all indicate platelet hyperactivation during SARS-CoV-2 infection. It was suggested that increased JAK3 expression and activity, which is upstream of mitogen-activated protein kinase (MAPK), plays a role in platelet activation in COVID-19 patients [64]. The role of MAPK in platelet hyperactivation in platelets derived from COVID-19 patients has also been reported in another study [65]. 

Further, critically ill COVID-19 patients have increased platelet–monocyte aggregation. Platelets derived from these patients were essential drivers of TF expression in the monocytes. In some patients, SARS-CoV-2 mRNA was detected in their platelets [65,66]. However, other studies found no ACE2 or TMPRSS2 on platelets or their precursor megakaryocytes and, thus, could not support the entry of SARS-CoV-2 in platelets [67,68]. It is conceivable that this controversy might be explained by the status of JAK signaling in cells, which is known to regulate the transcription and activation of ACE2 in the human airway epithelium [69,70]. 

## 6. A Controversial Role of JAK Signaling in Mediating the Effects of SARS-CoV-2

Emerging literature has uncovered another aspect of JAK–STAT regulation in patients with COVID-19 that is provocative in nature. In a wide array of cell lines, including A549 (human lung epithelial), Caco-2 (human colon epithelial), HuH-6 (human liver), AC-16 (human heart myocytes), SK-N-SH (human brain), HK-2 (human renal cortex and proximal tubule), and Vero (monkey kidney) infected with SARS-CoV-2, Chen et al. noted that SARS-CoV-2 downregulated JAK1, Tyk2, and the interferon receptor subunit 1 (IFNAR1), resulting in cellular desensitization to type I IFN signaling [71]. SARS-CoV-2 infection polyubiquitinated these proteins and induced their proteasomal degradation. In fact, the chemical inhibition of JAK kinases enhanced infection of stem cell-derived cultures, indicating that the virus benefited from an inhibited JAK–STAT pathway. In the same vein, other studies have shown that nonstructural proteins of SARS-CoV-2 inhibit STAT1 or STAT2 phosphorylation, and reduce the inflammatory conditions [72,73]. It is possible that some of the discrepancies concerning JAK–STAT signaling in COVID-19 depend on several factors, including the cell type tested in vitro vs. in vivo and the time of analyses post infection. Nonetheless, these studies call for exploring the potential therapeutic implications (see below).

## 7. JAK Inhibitors in the Treatment of COVID-19

Considering the documented contributions of JAK–STAT signaling to platelet and endothelial dysfunction in COVID-19, it was suggested that pharmacologic inhibition of this pathway might improve the overall inflammatory and vasculothrombotic manifestations of this disease [74]. It is noteworthy that JAK–STAT inhibitors are FDA-approved for other indications and can be repurposed for patients with COVID-19. 

**Figure 2:**
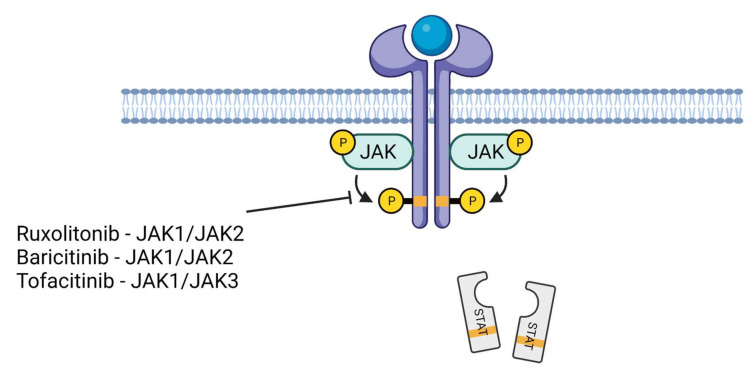
JAK–STAT inhibitors. Ruxolitonib and baricitinib inhibit JAK1/JAK2 while tofacitinib inhibits JAK1/JAK3. This event, in turn, suppresses the phosphorylation of STAT proteins and reduces their nuclear translocation. This phenomenon downregulates the inflammatory genes.

There is some degree of selectivity among JAK–STAT inhibitors. For example, baricitinib, or ruxolitinib inhibit JAK1 and JAK2, and tofacitinib inhibits JAK1 and JAK3 (Figure 2). Baricitinib inhibits JAK1/2 kinases by competing for the ATP needed for JAK1/2-induced activation by inflammatory cytokines such as IL-6. 

As baricitinib is approved for the treatment of rheumatoid arthritis with good efficacy and safety records, a pilot study was initiated to test its safety in combination with lopinavir–titonavir in patients with moderate COVID-19 pneumonia [75]. The study concluded that the drug administered for two weeks in 12 patients had no side effects and was considered safe. A double-blind, randomized, placebo-controlled trial evaluated the impact of time recovery from COVID-19 following treatment with baricitinib plus remdesivir. All the patients received remdesivir (≤10 days) and either baricitinib (≤14 days) (515 patients) or a placebo (control, 518 patients). Combination therapy was superior to remdesivir alone in reducing recovery time [28]. Another published study involving European cohorts showed that baricitinib administration reduced mortality by over 71% in 83 patients with moderate to severe SARS-CoV-2-induced pneumonia (median age of 81) [39]. An ongoing phase III clinical trial (NCT04421027) is a randomized interventional trial involving patients with mild COVID-19 (not needing ventilation) administered baricitinib daily for up to 28 days, showing 38% reduction in mortality by Day 28.

Ruxolitinib is an FDA- and European Union-approved JAK inhibitor for the treatment of myeloproliferative neoplasms (MPNs) involving JAK2 mutations [76]. Similar to baricitinib, several ongoing clinical trials aim at examining the benefit of ruxolitinib in treating COVID-19 patients. Encouraging data was obtained in a prospective, multicenter, single-blind, randomized controlled phase II trial involving patients with severe COVID-19. Forty-three patients were randomly assigned to receive ruxolitinib plus standard of care treatment (22 patients) or placebo based on standard of care treatment (21 patients). From the ruxolitinib group 90%, compared with about 62% from the control group, showed computed tomography (CT) improvement within two weeks. Further, the control group had about 14% one-month mortality, compared with 0% one-month mortality in the ruxolitinib group [77]. A multicenter phase II clinical trial (NCT04348071) in COVID-19 patients concluded that ruxolitinib was safe and effective in reducing incidence of multi-organ failure, leading to other phase III trial (NCT04362137) using ruxolitinib in COVID-19 patients with severe respiratory symptoms.

Tofacitinib predominantly inhibits JAK1 and JAK3 and is FDA-approved for treating rheumatoid arthritis, psoriatic arthritis, and ulcerative colitis. Tofacitinib was tested in a randomized, double-blinded, multicenter study with a placebo control involving 260 COVID-19 patients (NCT04469114) hospitalized with pneumonia and receiving standard of care therapy. The goal was to examine the effect on various clinical outcomes typical of COVID-19 [78]. Tofacitinib treatment for 28 days led to lower risk of death or respiratory failure in patients hospitalized with COVID-19 pneumonia, as compared with placebo-treated patients. Table 1 summarizes the status of clinical trials of JAK–STAT inhibitors in COVID-19.

There are several limitations of the ongoing clinical trials involving JAK–STAT inhibitors that warrant attention. Of note, these studies do not examine target cells such as platelets, nor do they examine vascular function, but rather focus on organ failure, inflammatory profile, and coagulopathy. While these are important clinical end points, mechanistic probing will help to uncover variability in responses to JAK–STAT inhibitors. As mentioned above, Chen et al. recently showed suppression of the proximal elements of the JAK–STAT pathway in a wide range of cultured cells infected with SARS-CoV-2. While these findings warrant human validation, they have potential therapeutic implications. For example, JAK–STAT inhibitors may not show their therapeutic benefit in scenarios where SARS-CoV-2 infection has already suppressed the JAK–STAT pathway in target cells. The status of the JAK/STAT pathway may drive responsiveness to JAK–STAT inhibitors, which underscores a need for biomarkers examining the activity of the JAK–STAT pathway in target cells. Such biomarkers can accompany the use of JAK–STAT inhibitors to yield maximum efficacy.

## 8. Conclusions

JAK–STAT signaling amplifies the pathologic effects of SARS-CoV-2 infection in various cells and is implicated in widespread thrombosis, contributing to catastrophic end-organ failure. A wealth of studies provides a strong rationale for the use of JAK inhibitors in improving COVID-19 outcomes. While waiting for their results, caution is in order, as these inhibitors may have variable responses, or may paradoxically exacerbate pathologic manifestations of SARS-CoV-2 infection [71]. More studies are needed to identify the kinetics of JAK–STAT activation in cells involved in regulating thrombosis, and the impact of JAK–STAT inhibition on inflammatory and thrombotic manifestations in COVID-19 patients.

## Figures and Tables

**Table 1:** JAK–STAT inhibitors in the treatment of COVID-19.

Inhibitor Used/JAK Selectivity	Approval for Other Diseases	End Point/Treatment Duration/Site	Clinical Trial or Reference Number	Outcome
baricitinib/JAK1 or JAK2	FDA- and EU-approved for rheumatoid arthritis, EU-approved for atopic dermatitis	COVID-19 recovery time/14 days/USA	clinical trial number: NCT04401579	baricitinib with remdesivir was superior to remdesivir alone in reducing recovery time
		reduced mortality in moderate to severe COVID-19 pneumonia/14 days/Europe	reference: [39]	baricitinib administration reduced mortality by over 71%
		recovery time in mild COVID-19 (not requiring mechanical ventilation)/28 days/USA	clinical trial number: NCT04421027 (https://investor.lilly.com/news-releases/news-release-details/lilly-and-incyte-announce-results-phase-3-cov-barrier-study (accessed on 30 December 2021))	38% reduction in mortality by day 28 in patients treated with baricitinib in addition to corticosteroids and remdesivir
ruxolitinib/JAK1 or JAK2	FDA- and EU-approved for myelofibrosis and polycythemia vera, FDA-approved for graft-versus-host disease	chest CT improvement and mortality/14 days/China	reference: [77]	chest CT improvement within 14 days of treatment
		multi-organ failure/14 days/USA	vlinical trial number: NCT04348071	less incidence of organ failure upon treatment, leading to an ongoing phase III clinical trial (NCT04362137)
tofacitinib/JAK1 or JAK3	FDA- and EU-approved for rheumatoid arthritis, psoriatic arthritis, and ulcerative colitis	respiratory failure and death in patients hospitalized with COVID-19 pneumonia/28 days/USA	vlinical trial number: NCT04469114	lower risk of death or respiratory failure as compared to placebo-treated patients
